# A Face-Aging App for Smoking Cessation in a Waiting Room Setting: Pilot Study in an HIV Outpatient Clinic

**DOI:** 10.2196/10976

**Published:** 2018-08-15

**Authors:** Titus Josef Brinker, Christian Martin Brieske, Stefan Esser, Joachim Klode, Ute Mons, Anil Batra, Tobias Rüther, Werner Seeger, Alexander H Enk, Christof von Kalle, Carola Berking, Markus V Heppt, Martina V Gatzka, Breno Bernardes-Souza, Richard F Schlenk, Dirk Schadendorf

**Affiliations:** ^1^ National Center for Tumor Diseases Department of Translational Oncology German Cancer Research Center Heidelberg Germany; ^2^ Department of Dermatology Heidelberg University Hospital University of Heidelberg Heidelberg Germany; ^3^ Department of Dermatology Essen University Hospital University of Duisburg-Essen Essen Germany; ^4^ German Cancer Consortium University of Heidelberg Heidelberg Germany; ^5^ Cancer Prevention Unit German Cancer Research Center Heidelberg Germany; ^6^ Section for Addiction Medicine and Addiction Research University Department of Psychiatry and Psychotherapy University Hospital Tübingen Tübingen Germany; ^7^ Department of Psychiatry and Psychotherapy Ludwig Maximilian University of Munich Munich Germany; ^8^ Universities of Giessen and Marburg Lung Center Department of Internal Medicine Justus-Liebig-University Gießen Germany; ^9^ Department of Dermatology University Medical Center Munich University of Munich Munich Germany; ^10^ Department of Dermatology and Allergic Diseases University of Ulm Ulm Germany; ^11^ School of Medicine Federal University of Ouro Preto Ouro Preto Brazil; ^12^ Trial Center National Center for Tumor Diseases German Cancer Research Center Heidelberg Germany

**Keywords:** face aging, smoking cessation, HIV, mobile apps, HIV patients, HIV seropositivity, smoking, cessation, tobacco smoking, morphing

## Abstract

**Background:**

There is strong evidence for the effectiveness of addressing tobacco use in health care settings. However, few smokers receive cessation advice when visiting a hospital. Implementing smoking cessation technology in outpatient waiting rooms could be an effective strategy for change, with the potential to expose almost all patients visiting a health care provider without preluding physician action needed.

**Objective:**

The objective of this study was to develop an intervention for smoking cessation that would make use of the time patients spend in a waiting room by passively exposing them to a face-aging, public morphing, tablet-based app, to pilot the intervention in a waiting room of an HIV outpatient clinic, and to measure the perceptions of this intervention among smoking and nonsmoking HIV patients.

**Methods:**

We developed a kiosk version of our 3-dimensional face-aging app Smokerface, which shows the user how their face would look with or without cigarette smoking 1 to 15 years in the future. We placed a tablet with the app running on a table in the middle of the waiting room of our HIV outpatient clinic, connected to a large monitor attached to the opposite wall. A researcher noted all the patients who were using the waiting room. If a patient did not initiate app use within 30 seconds of waiting time, the researcher encouraged him or her to do so. Those using the app were asked to complete a questionnaire.

**Results:**

During a 19-day period, 464 patients visited the waiting room, of whom 187 (40.3%) tried the app and 179 (38.6%) completed the questionnaire. Of those who completed the questionnaire, 139 of 176 (79.0%) were men and 84 of 179 (46.9%) were smokers. Of the smokers, 55 of 81 (68%) said the intervention motivated them to quit (men: 45, 68%; women: 10, 67%); 41 (51%) said that it motivated them to discuss quitting with their doctor (men: 32, 49%; women: 9, 60%); and 72 (91%) perceived the intervention as fun (men: 57, 90%; women: 15, 94%). Of the nonsmokers, 92 (98%) said that it motivated them never to take up smoking (men: 72, 99%; women: 20, 95%). Among all patients, 102 (22.0%) watched another patient try the app without trying it themselves; thus, a total of 289 (62.3%) of the 464 patients were exposed to the intervention (average waiting time 21 minutes).

**Conclusions:**

A face-aging app implemented in a waiting room provides a novel opportunity to motivate patients visiting a health care provider to quit smoking, to address quitting at their subsequent appointment and thereby encourage physician-delivered smoking cessation, or not to take up smoking.

## Introduction

There is strong evidence for the effectiveness of addressing tobacco use in health care settings [1-11]. However, few smokers receive cessation advice when visiting a hospital [12,13] which is caused by many different reasons [14] and is therefore difficult to change.

Face-aging interventions, in which a photograph of the user is altered to predict the user’s future appearance, have been shown to motivate healthier behavioral choices in adiposity prevention, skin cancer prevention, and smoking cessation settings [15-35]. These preliminary results can be explained by the high importance of appearance for a person’s self-concept, particularly during adolescence [36].

However, to the best of our knowledge, the only completed prospective randomized trial to investigate the effectiveness of a face-aging intervention on actual behavior (smoking) was that of Burford et al [37]. Burford and her team recruited 160 participants (80 allocated to the control group and 80 to the intervention group) from 8 metropolitan community pharmacies located around Perth city center in Western Australia. All the participants received standardized smoking cessation advice, but those in the intervention group were also digitally photoaged by the internet-based APRIL Face Aging software to show images of what they might eventually look like as a lifelong smoker and as a nonsmoker. At the 6-month follow-up, 5 (6%) of the 80 control group participants suggested they had quit smoking, although this was confirmed by carbon monoxide validation in only 1 of them. In contrast, 22 (27%) of the 80 intervention group participants reported quitting, with 11 confirmed by carbon monoxide testing, a statistically significant difference in confirmed quitting (χ^2^_1_=9.0; *P*=.003; test power=80%). However, the study had several limitations: the photographs now appear technologically outdated, they were taken in an over-the-counter setting that always required the time of another person, they were not available for free, and the approach did nothing to address the poor initiation of smoking cessation by doctors as recommended by guidelines [38].

We have developed a 3-dimensional face-aging, tablet-based app, Smokerface, that alters a self-taken image of the user’s face to simulate what the user would look like in 1 to 15 years’ time as either a smoker or a nonsmoker [39]. In this study, we hypothesized that hospital waiting rooms provide an effective setting for encouraging smoking cessation via the app because this would allow most patients visiting a health care provider to be passively exposed without the need for preluding action by health care personnel. To the best of our knowledge, no previous interventions in the field have implemented new technology for behavioral change in waiting rooms. We chose an HIV outpatient clinic for piloting our intervention, because HIV-positive patients are approximately twice as likely to smoke as the general population [40-45], ensuring that a comparably high number of the sample exposed to the intervention would be current smokers.

Therefore, the aim of this study was to develop an intervention that would make use of the waiting time of patients for smoking cessation by exposing them to the face-aging app and to measure the perceptions of smokers and nonsmokers after using the app.

## Methods

### Ethical Considerations

We planned this study at the University Hospital of Essen in Germany in early 2017 and implemented it in October 2017. All the participants were adults, and participation in the intervention and questionnaire survey was voluntary. The questionnaire was anonymous, and no personal data were stored. All images were instantly deleted automatically by the kiosk version of the app. We considered oral consent to be sufficient for participation in the survey. Before participants could use the app, they were informed about the screen-mirroring procedure by an information board placed adjacent to the tablet. The ethics committee of the Essen University Hospital, Essen, Germany, approved the study.

### Experimental Setup

**Figure 1:**
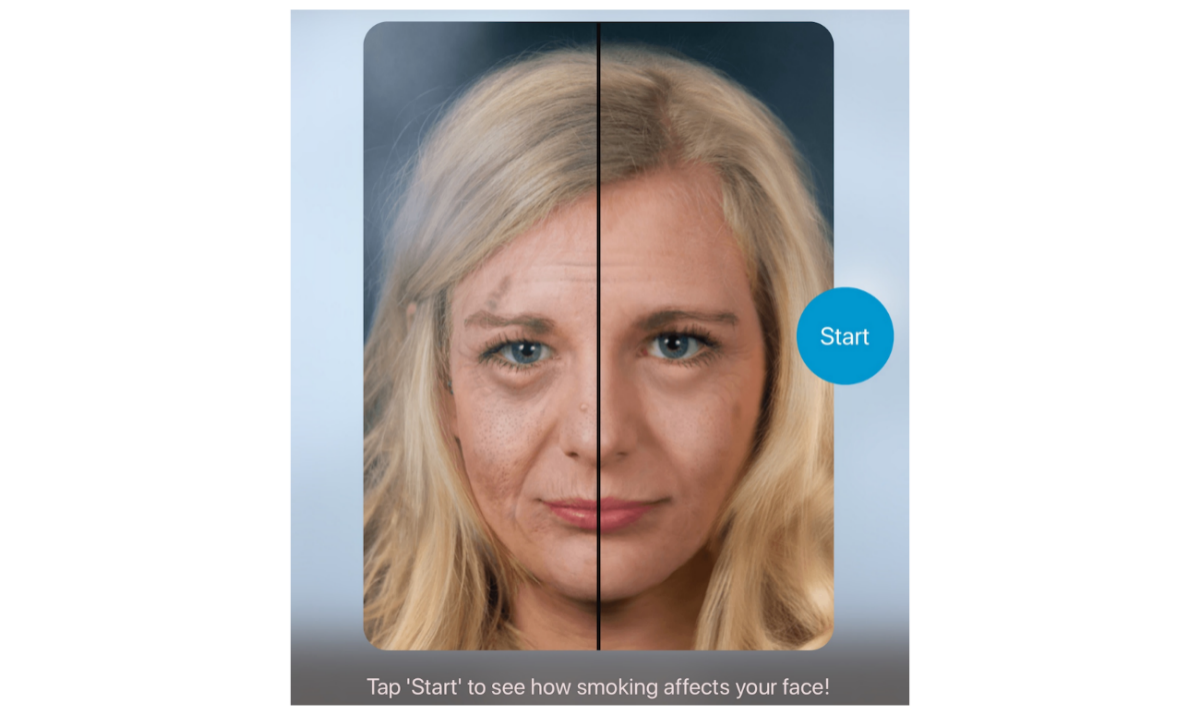
Start screen of the kiosk version of our face-aging app Smokerface, running on an Apple iPad (iOS).

**Figure 2:**
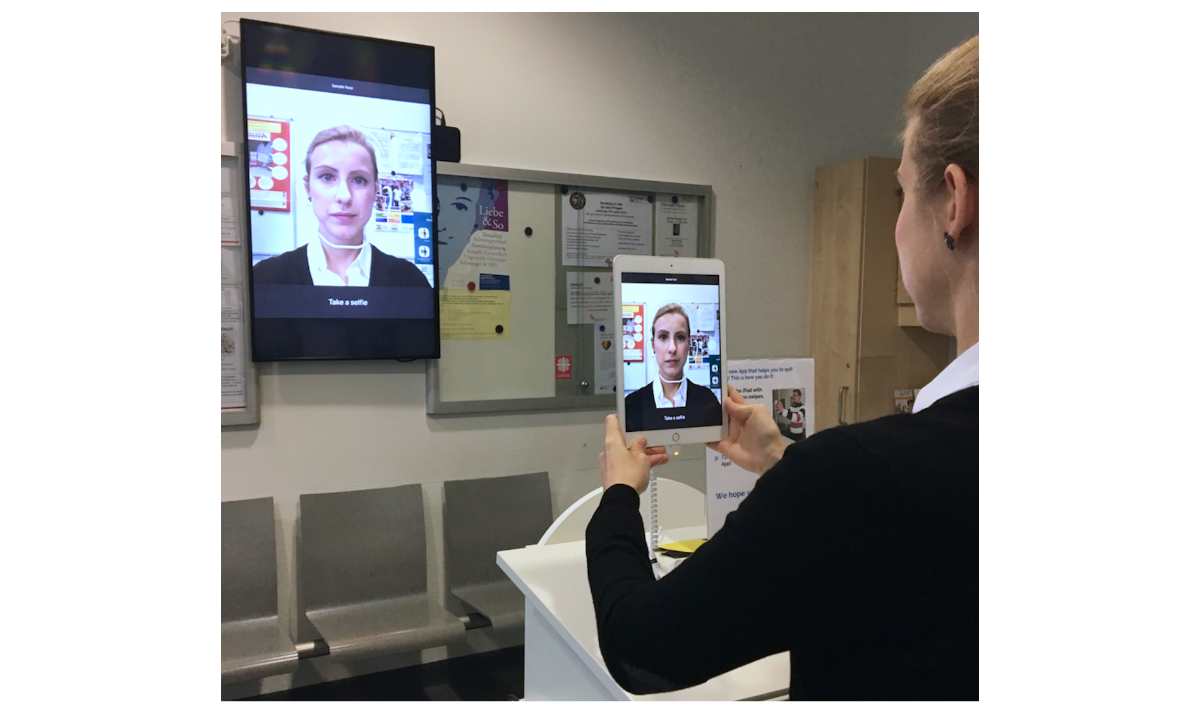
Setup of the smoking cessation face-aging intervention in the waiting room of our HIV outpatient clinic. After the home screen, users were instructed to “Tap ‘Start’ to see how smoking affects your face!” The original setup was in German.

We developed a kiosk version of the Smokerface app (Figure 1). We placed an Apple iPad (iOS) tablet (Apple Inc, Cupertino, CA, USA) on which this version of the app was running on a table in the middle of the waiting room of our HIV outpatient clinic and connected it to a large monitor attached to the opposite wall, which mirrored the screen of the iPad (Figure 2). An explanatory note was displayed on a board next to the tablet.

**Figure 3:**
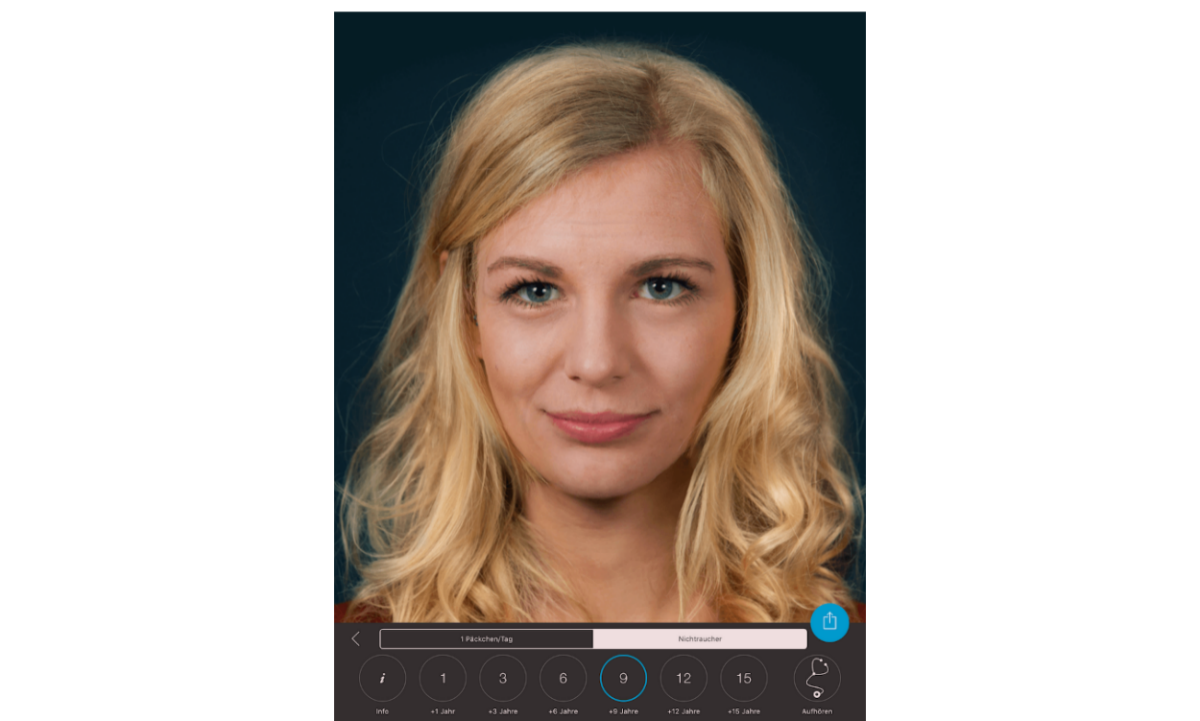
Example image of the user simulating how she might look after 9 years of aging without smoking. The screenshot was taken directly from the iPad.

**Figure 4:**
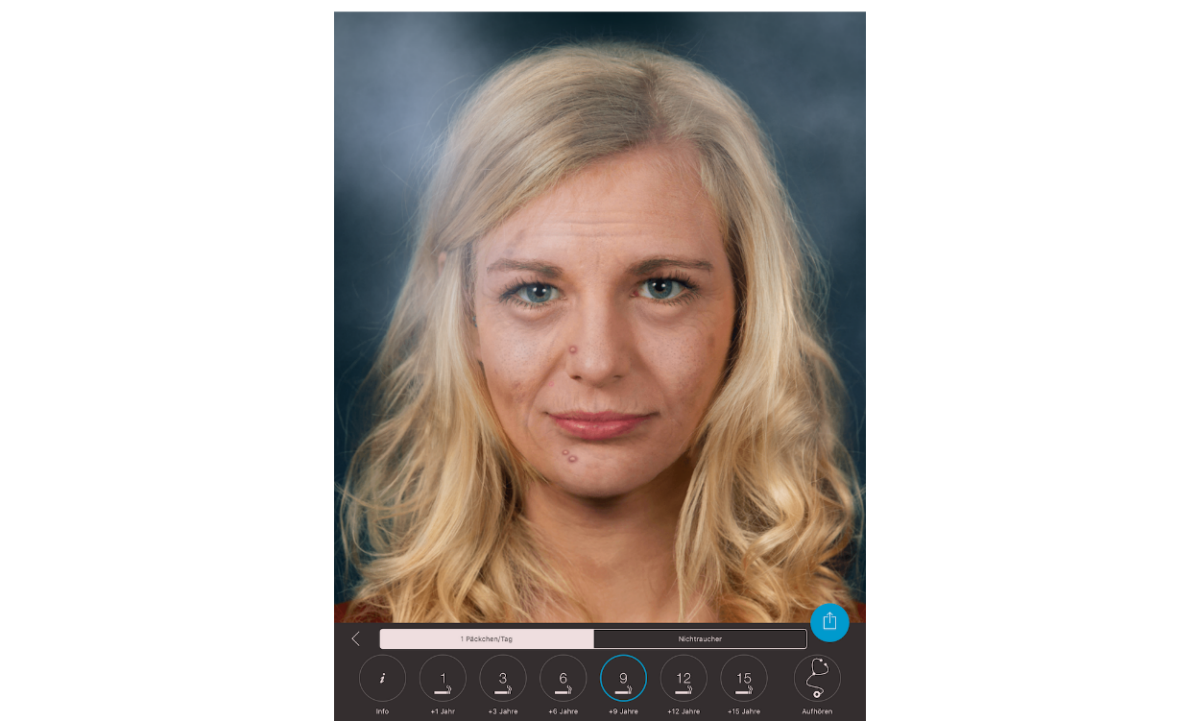
Example image of the user simulating how she might look after 9 years of aging with smoking a pack of cigarettes a day. The screenshot was taken directly from the iPad.

The tablet screen displayed the instruction “Tap ‘Start’ to see how smoking affects your face!” (written in German) and was in guided access mode to ensure that patients could not quit the app. The app then displayed images of the patient’s face simulating their appearance after 1 to 15 years of not smoking (Figure 3) or smoking (Figure 4).

### Procedure

A researcher counted all the patients who visited the waiting room, noted their sex, and timed their total time spent in the waiting room. If a patient did not try the app within 30 seconds of starting their wait, the researcher encouraged the patient to use the app, following a standardized protocol. Patients who used the app were then asked whether they were smokers or nonsmokers and were asked to complete, voluntarily, the appropriate one of 2 paper-and-pencil questionnaires. Both smoking status–specific questionnaires captured the age and sex of the participant, as well as the participant’s perceptions of using the app, on 4-point Likert scales (from “absolutely true” to “absolutely false”). In addition, the participant was asked about the number of other patients in the room during the use of the app, the reactions of the other patients, and how the participant perceived those reactions (on 4-point Likert scales).

The smokers’ questionnaire additionally included 2 standard Fagerström items to calculate the Heaviness of Smoking Index (HSI), a validated measure of smoking dependence that has also been shown to predict quit success [46]: “How many cigarettes do you smoke per day?” and “When do you smoke the first cigarette after waking up?” Prior to the study, we tested the questionnaires in a small subsample of 32 patients to ensure that the questions were understandable and to measure the time needed to complete them (approximately 4 minutes).

### Data Analysis

We performed descriptive analysis of data with IBM SPSS Statistics version 25 (IBM Corporation). We undertook no tests for significance due to the explorative nature of the study.

## Results

### Sample Characteristics

The sample consisted of 464 patients (male: 355/464, 78.7%), of whom 179 filled out a questionnaire (male: 79.0%; median age 42 years; range 23-76 years). Of the 179 patients who completed the questionnaire, 84 (46.9%) smoked; of the smokers, 66/82 (80.5%) were men and 16/82 (19.5%) were women (2 smokers did not indicate their sex).

Among the 84 smokers, 25/83 (30%) had a low HSI, 43/83 (52%) had a medium HSI, and 15/83 (18%) had a high HSI. One participant did not answer both Fagerström items; therefore, we could not calculate the HSI for that person.

### Participation

**Figure 5:**
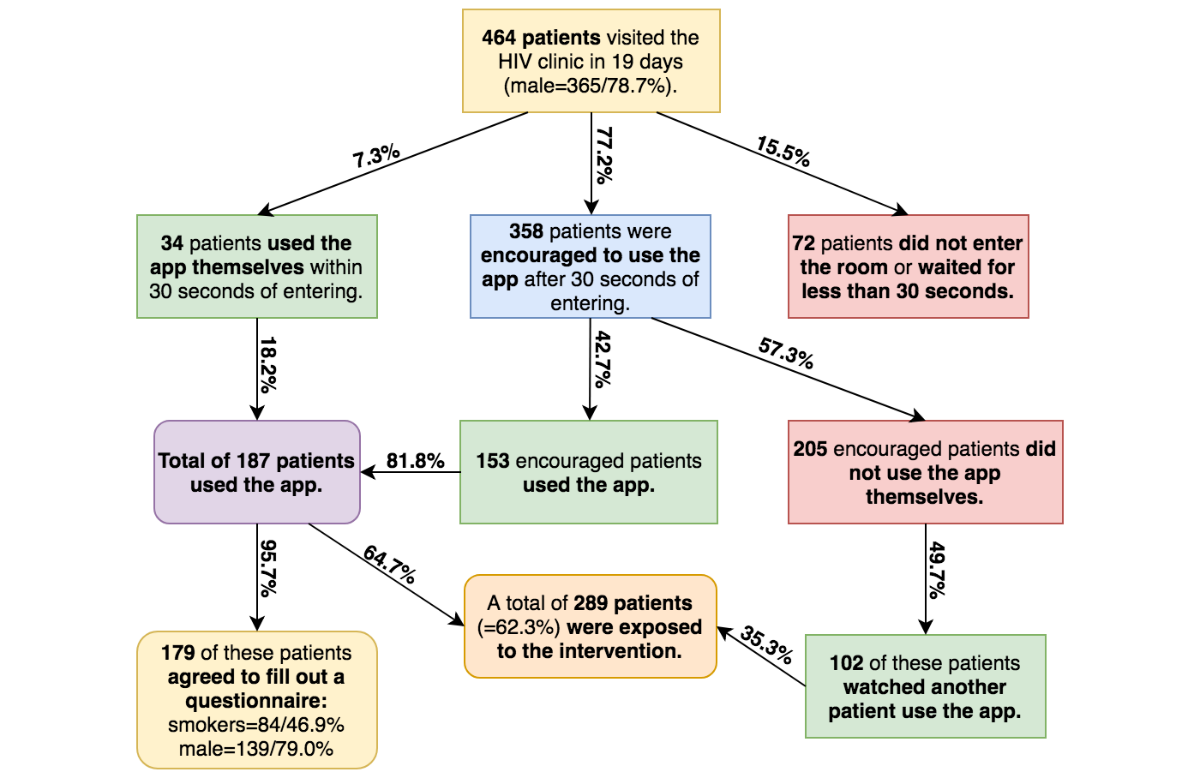
Levels of participation, sex, and smoking status of the waiting room visitors.

The intervention was implemented in our waiting room for 19 days, and Figure 5 illustrated study participation. The average waiting time for all patients was 21 minutes.

### Perceptions About the Intervention

Among the 84 smokers, 55 of 81 (68%) reported that the intervention motivated them to quit (men: 45, 68%; women: 10, 67%, with 3 smokers not answering this question), 41 (51%) reported that it motivated them to discuss quitting with their doctor (men: 32, 49%; women: 9, 60%), and 72 (91%) perceived the intervention as fun (men: 57, 90%; women: 15, 94%). Of the nonsmokers, 92 of 94 (98%) reported that it motivated them to never take up smoking (men: 72, 99%; women: 20, 95%).

### Other Patients in the Waiting Room

The numbers of other patients in the waiting room at the time a participant tried the app were as follows: no other patients, 30 (17%) cases; 1 to 3 other patients, 86 (49%) cases; 4 to 6 other patients, 56 (32%) cases; 7 to 10 other patients, 3 (2%) cases; 11 or more patients, 1 (0.6%) case.

Table 1 summarizes the participants’ descriptions of the reactions of the other patients in the waiting room (in answer to the question “How did the other people in the room react to your public selfie?”). In a considerable proportion of cases (48/132, 36.4%), 1 or more of the other patients reacted by trying the app themselves.

**Table 1:** Reactions of other patients in the waiting room when a participant tried the app (only the cases where there was at least one other patient in the waiting room).

Patient group		Others tried the app themselves, n (%)		Quitting was a topic of discussion afterward, n (%)		They encouraged me to quit or stay a nonsmoker, n (%)		They were astonished, n (%)			Their reactions were very strong, n (%)		
**All patients**		**N=132**		**N=138**		**N=126**		**N=132**			**N=131**		
	False/absolutely false	84 (63.6)		61 (44.2)		70 (55.6)		81 (61.4)			90 (68.7)		
	True/absolutely true	48 (36.3)		77 (55.8)		56 (44.4)		51 (38.6)			41 (31.3)		
**Smokers**		**N=63**		**N=64**		**N=77**		**N=64**		**N=60**		
	False/absolutely false	40 (63)		23 (36)		24 (41)		39 (61)			42 (70)		
	True/absolutely true	23 (36)		41 (64)		53 (59)		25 (39)			18 (30)		
**Nonsmokers**		**N=69**		**N=74**		**N=67**		**N=68**		**N=71**		
	False/absolutely false	44 (64)		38 (51)		46 (69)		42 (62)			48 (68)		
	True/absolutely true	25 (36)		36 (49)		21 (31)		26 (38)			23 (32)		

**Table 2:** Participants’ perceptions of other patients’ reactions to the participant’s use of the app (only the cases where there was at least one other patient in the waiting room).

Patient group		Motivated me to quit or remain a nonsmoker, n (%)	Helpful, n (%)	They gave me quitting advice, n (%)	There were no reactions, n (%)
**Smokers**		**N=49**	**N=47**	**N=42**	**N=15**
	False/absolutely false	19 (48)	14 (30)	19 (45)	N/A^a^
	True/absolutely true	30 (61)	33 (70)	23 (55)	15 (100)
**Nonsmokers**		**N=51**	**N=42**	**—**	**N=19**
	False/absolutely false	11 (22)	15 (36)	N/A	N/A
	True/absolutely true	40 (78)	27 (64)	N/A	19 (100)

^a^N/A: not applicable.

In most cases (77/138, 55.8%), the participant’s use of the app initiated a discussion on quitting in the waiting room; this was even more the case (41/64, 64%) when the participant was a smoker. In addition, 59% (53/77) of the participants were encouraged to quit by the other patients in the waiting room after using the app, which appeared to be often accompanied by quit advice (23/42, 55%; Table 2).

Table 2 summarizes how the participants perceived the reactions of the other patients, answering the question “How did you perceive those reactions?” The reactions were largely perceived as helpful (by 33/47, 70% of smokers and 27/42, 64% of nonsmokers). Indeed, the reactions provided motivation for 61% (30/49) of the smokers to quit, with advice on quitting offered to 55% (23/42) of the smokers.

## Discussion

### Principal Findings

The face-aging app setup was successful in exposing the majority of patients visiting our HIV outpatient clinic to a smoking cessation intervention. The face-aging procedure itself and the public nature of the face-aging procedure that triggered reactions of other waiting patients were perceived as motivating to quit smoking and helpful by the majority of smoking as well as nonsmoking patients.

To the best of our knowledge, this is the first study to implement new technology in a waiting room in order to use the patients’ waiting time to encourage smoking cessation. The results suggest a huge potential for the large-scale exposure of patients visiting health care providers to the technology. The effectiveness of this study remains subject to further study, and we aim to test the effectiveness in promoting smoking cessation in a randomized controlled trial. Further long-term studies should also examine the effects of group interactions and changes in the subjective norm because of setting the intervention in a waiting room.

Patients who are positive for HIV are approximately twice as likely to smoke as the general population (46.9% of our sample were smokers, compared with 23.9% in the general population) [40-45]. Effective interventions for this patient group remain scarce [4,47-55], and the population-attributable risk of death associated with smoking is double that of the general population [56]. In countries such as Germany where HIV care is well organized and antiretroviral therapy is free of charge, HIV-infected smokers lose more life-years to smoking than to HIV [56]. Hence, while our intervention is not specifically tailored to any patient group and could be applied in any patient waiting room, an HIV outpatient clinic provided an ideal setting for piloting the intervention.

The nonadherence of physicians to smoking cessation guidelines is a situation that is prevalent in many countries because of role incongruence and a lack of time, financial reimbursement, and appropriate training [14,38]. Being a physician can be an extremely stressful occupation in modern times, with increases in the level of bureaucracy required and the number of patients per doctor. Interventions that help a physician identify and motivate smokers willing to quit have the potential to increase population health and thereby reduce the workload for the medical profession, helping in the fight against tobacco-attributable diseases. Our approach shows promise as a possible simple solution to help physicians meet their smoking cessation obligations without placing too great a burden on them. However, our results also point to limitations and raise questions that need to be addressed in future research.

### Initiation of the Use of the App

In this study, the researcher intervened by inviting patients to try the app if they did not do so spontaneously within 30 seconds of starting their wait in the waiting room. We decided on this approach for 3 reasons: (1) the HIV waiting room is particularly busy, with an average waiting time of only 21 minutes; (2) the number of patients waiting there tends to be low, with just 24 patients per day on average between 7:30 AM and 4:45 M; and (3) HIV patients tend to be rather shy in health care settings, as described by the experienced head of our HIV outpatient clinic. Nevertheless, 34 (7.3%) of the 464 patients tried the app within 30 seconds without prompting, indicating the likelihood of successful passive exposure with more time or in settings with higher patient density and longer waiting times. However, it is possible that other patient groups are even more reluctant to have their photograph transmitted to a publicly visible screen, and there might be barriers to the use of such a technology, especially with older patients. In this study, 48 of 132 (36%) participants reported that another patient tried the app straight after seeing them use it, which further strengthens the hypothesis that there would be less need of external prompting in fuller waiting rooms.

### Short- and Longer-Term Implications

We received no complaints about patients feeling bullied, according to the physicians who worked in our outpatient clinic at the time of the study, and a great majority of the smokers perceived the intervention as fun. However, the question of feeling bullied could be addressed more explicitly in future research because of the nature of the intervention. We observed that the intervention resulted in interaction between patients where there had been none. Usually, patients waiting in this waiting room sit silently using their mobile phones, reading a newspaper, or just staring at the ground. When the intervention was implemented, patients began talking to each other about the intervention and smoking cessation, finding a common topic they could discuss. Our researcher reported that the overall atmosphere of these conversations was encouraging and positive, and this was reflected in the questionnaire data. Those who had already quit smoking shared their advice and even encouraged addressing the topic at the participant’s subsequent appointment; this was reported by 19 of 42 (45%) of the smokers in the questionnaire. In addition, 41 of 81 (51%) of the smokers reported that the intervention itself motivated them to address the topic at their upcoming appointment. Future studies should obtain information from the physicians treating those patients about whether smoking cessation was raised. Following this study, clinicians reported an increased rate of questions on how to quit, but this was not recorded in an objective fashion.

According to our data, 48 of 132 (36.4%) patients tried the app immediately after watching another person do it. In addition, it is reasonable to speculate that simply watching the intervention and perhaps engaging in a conversation arising from it in a full waiting room would motivate a patient to start quitting smoking due to a potential change in their subjective norm [57].

### Projection of Potential Effects

During the 19 days of the study, a total of 289 patients in 1 waiting room used or were exposed to the smoking cessation app. This is equivalent to approximately 5500 patients per waiting room per year or to 176,000 patients per year if implemented in all 32 waiting rooms at our hospital. If we assume that the prospective effects measured by Burford et al (that 21.2% of smokers aged 18 to 30 years quit following the use of a similar method [37]) can be transferred to our intervention and that the prevalence of smoking among this hospital’s patients is approximately 30%, then approximately 11,000 smoking patients would quit per year. In our sample, the median age was 42 years, meaning that 9 life-years would be saved per patient on average, equivalent to 99,000 saved life-years in total each year [58]. The total cost was US $1500 for 1 waiting room, equivalent to US $0.48 per saved life-year. However, transferability has not been proven, and the prospective effects might be weaker for older patients. In our sample, just 22 (12.3%) of the 179 participants who completed the questionnaire were aged 18 to 30 years. Thus, if the intervention had no effect at all for any patient other than those aged 18 to 30 years, the cost per saved life-year would be US $3.90, 1.320 patients would quit, and 13,200 life-years would be saved per year of implementation.

As for any smoking cessation intervention that has not yet been evaluated in large randomized trials, health systems and insurance companies may be hesitant to reimburse clinics for implementation of this technology. Funding opportunities for health care providers will improve with prospective research on the technology’s influence on smoking behavior.

### Study Limitations

This study had several limitations. Many of the participants were called by the nurse while still completing the questionnaire. We anticipated this problem and put the questions about individual perceptions of the intervention and important sociodemographic data and smoking status at the start of the questionnaire. The loss of data for these initial items was relatively low. Our study reported only cross-sectional data, and we could only estimate the influence on actual behavior. However, behavioral predictors, such as the behavioral intention to perform a certain behavior, indicate effectiveness in accordance with the theory of planned behavior [57]. In addition, although anonymity decreases social desirability bias, the participants completing the questionnaires may nevertheless have felt pressure to answer in a socially desirable way because the researcher was present in the room. To minimize this, the participants were left to themselves for completing the questionnaire, which they could then drop into a sealed box to further reduce the risk of bias.

### Other Studies That Help Physicians Identify Unhealthy Behaviors

It should be noted that other eHealth interventions can be found in the literature that at least help physicians to identify unhealthy behaviors of patients while only indirectly influencing that behavior [59-76]. These mostly comprise digitized screening and early detection tools. The majority of this work focuses on mental health or the prediction of mental disease [59,64-66,68,69,74,77], and only a few publications have focused on predictors of chronic disease in general, including substance abuse [63,65,68,70,77]. However, helping physicians to identify smokers is only one aim of the intervention presented here; we think it is at least likewise important to investigate its direct effect on quitting behavior in future studies.

### Conclusion

The use of a face-aging smoking cessation app in waiting rooms provides a new, enjoyable opportunity to motivate the majority of smokers visiting a health care provider to quit smoking or to address quitting at their subsequent appointment and nonsmokers to never take up smoking. It thereby facilitates physician-delivered smoking cessation. We plan a cluster-randomized trial of the app in 10 waiting rooms. This will focus on long-term smoking abstinence rates, analyzing the impact on different patient subgroups and the interplay of waiting times and modes of initiation. In addition, we plan to repeat the experiment using the Sunface skin cancer awareness app [25] to determine if it shows similar promise.

## Abbreviations

HSI: Heaviness of Smoking Index
